# Toxicity evaluation of an essential oil mixture from the Cretan herbs thyme, Greek sage and Cretan dittany

**DOI:** 10.1038/s41538-020-00080-1

**Published:** 2020-11-09

**Authors:** Konstantina Kalyvianaki, Panagiotis Malamos, Niki Mastrodimou, Ioanna Manoura-Zonou, Rodanthi Vamvoukaki, George Notas, Niki Malliaraki, Eleni Moustou, Maria Tzardi, Stergios Pirintsos, Christos Lionis, George Sourvinos, Elias Castanas, Marilena Kampa

**Affiliations:** 1 Laboratory of Experimental Endocrinology, School of Medicine, University of Crete, Heraklion, Greece; 2 Laboratory of Pharmacology, School of Medicine, University of Crete, Heraklion, Greece; 3 Laboratory of Clinical Chemistry and Biochemistry, University Hospital of Heraklion, Crete, Greece; 4 Laboratory of Pathology, School of Medicine, University of Crete, Heraklion, Greece; 5 Department of Biology, University of Crete, 71409 Heraklion, Greece; 6 Botanical Garden, University of Crete, 74100 Rethymnon, Greece; 7 Clinic of Social and Family Medicine, School of Medicine, University of Crete, Heraklion, Greece; 8 Laboratory of Clinical Virology, School of Medicine, University of Crete, Heraklion, Crete Greece

**Keywords:** Signs and symptoms, Diseases

## Abstract

The importance of herbal extracts on health, which was initially based on ethnopharmacological and traditional knowledge, becomes increasingly well documented by numerous experimental and intervention studies. The daily use of beverages from different aromatic plants which becomes more popular nowadays, has been a tradition in Crete, and a habit that has been linked to the longevity seen in the island. Additionally, a certain combination of aromatic plants has been used against common cold and influenza. Interestingly, when such a mixture of essential oils from Cretan herbs (Cretan Aromatic Plants essential oil, CAPeo, from thyme, Greek sage, and Cretan dittany) was formulated, significant antiviral properties were observed in vitro and a significant reduction in the duration and severity of symptoms of patients with upper respiratory tract infections was found in a clinical study. However, since many plants extracts can exert toxic effects, toxicity issues should be properly addressed. In the present work we present an acute and sub-chronic toxicity evaluation for this mixture of aromatic plants’ essential oils in rats. In fact, it is the only toxicity study for Cretan dittany. We report absence of toxicity, rendering the use of the mixture of essential oils from Cretan dittany, Greek sage and thyme as safe.

## Introduction

Extensive research, in recent years, has revealed the pivotal role of oxidative stress in several chronic diseases, such as diabetes, cardiovascular, autoimmune, neurodegenerative and cancer, and the importance of antioxidants, including polyphenols. As a result, there is a great interest in natural antioxidative compounds which are widely found in plant material. Herbal beverages, being an important source for such compounds, such as polyphenols, which possess antioxidant, antiviral, and anti-inflammatory activities^1–3^, have gained popularity among health-conscious consumers.

The daily use of beverages from different aromatic plants has been a tradition of Mediterranean and Southeastern populations, including Cretans. In fact, the longevity that was observed in the island of Crete (Salehi^4^ and references herein) can be partially attributed to the use of herbal extracts. This assumption guided a laboratory research certain years ago^5^, after an observational research that addressed morbidity and mortality data from a rural population in Crete^6,7^. Moreover, there were evidence pointing out that certain combinations of different aromatic plants of Crete could be beneficial for the prevention and cure of the common cold and influenza^5^. Based on these, our group recently has formulated a mixture of essential oils from three aromatic plants of Crete, thyme or Spanish oregano (*Thymbra capitata* (L.) Cav.), dictamnus or Cretan dittany (*Origanum dictamnus* L.,) and Greek sage (*Salvia fruticosa* Mill.; Cretan Aromatic Plants essential oil, CAPeo). We performed an in vitro study that verified the antiviral potential of CAPeo against a wide range of upper respiratory tract viruses. Our results showed that CAPeo had a remarkable antiviral activity against influenza A/H1N1 virus strains, influenza B, and human rhinovirus 14 (HRV14), mainly due to defective trafficking of influenza A Nucleoprotein^8^. Additionally, in a double-blind randomized controlled trial, where CAPeo was administered to human subjects at doses corresponding to the consumption of 2–3 cups of beverage per day, confirmed that CAPeo decreases symptoms duration and severity, as well as systemic inflammation, as assayed by C-reactive protein [CRP] levels^9,10^, in patients with upper respiratory tract infections, with an apparent absence of toxicity, after the recommended 1-week consumption. Having in mind that certain herbal essential oils may have toxic effects, and that there are limited data concerning the toxicity of the used aromatic plant essential oils as a mixture, a thorough investigation of in vivo toxicity was essential, in spite of the lack of evidence for toxicity in humans^9^. Apparently, no such data were available for CAPeo, while there were only few reports for only two out of the three aromatic plants used in CAPeo, separately. Thyme essential oil, as well as some of its major constituents such as thymol and carvacrol has been the most studied. However, the majority of studies have examined *Thymus vulgaris* and not *Coridothymus capitatus* that is included in CAPeo (see Salehi et al.^11^ for a review, and Rojas-Armas et al.^12^). Nevertheless, it was found that *Thymus vulgaris* essential oil is less toxic than its essential constituent thymol. As reported by EFSA^13^ thymol has a moderate acute oral toxicity and high doses should be avoided (LD_50_ 1000 mg/kg bw)^12^. As far as Greek sage (*Salvia fruticosa*) is concerned no data are available. There are few data only for *Salvia officinalis* reporting unwanted effects such as salivation, vomiting, tachycardia, and vertigo only after prolonged use and in overdose cases (ethanolic extract or oil corresponding to more than 15 g of the leaves)^14^, and demonstrating a slight oral acute toxicity in rats^15^. Finally, no toxicity studies are available for dittany.

Therefore, the aim of the present work was to evaluate acute and sub-chronic toxicity of the essential oils from the three aforementioned aromatic plants, as a mixture (CAPeo), in a rat model. Different doses (at the range that CAPeo is effective, as an antiviral agent and we have previously used in the clinical study^9^) were tested by assaying several biochemical markers and histological analysis of selected organs for signs of necrosis, injury, or inflammation. Our findings clearly show that CAPeo does not induce any kind of toxicity in its either short- or long-term administration, even after administration of 20x the suggested dose for humans.

## Results

### Acute toxicity study

During the 24 h and 15 days of intervention there were no visible signs of toxicity (such as tremors, numbness, salivation, or diarrhea). All animals survived and no behavioral changes were observed.

**Fig. 1:**
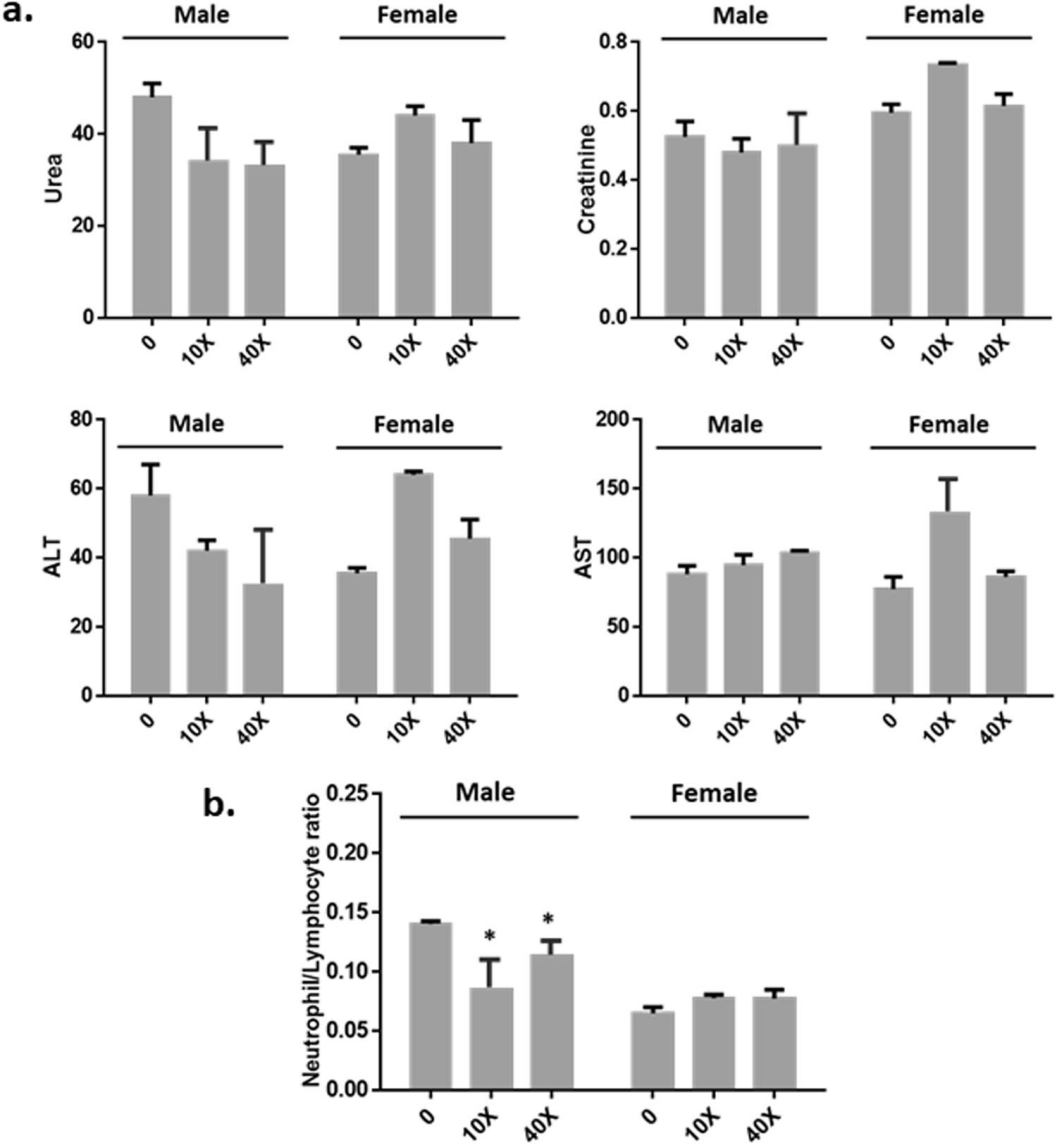
Acute effect of CAPeo-Changes 24 h after treatment. The effect of CAPeo on **a** creatinine (mg/dl), urea (mg/dl), AST(U/L), and ALT(U/L) levels, and **b** the neutrophil to lymphocyte ratio was examined 24 h after treatment. Two different CAPeo doses were tested: 10X and 40X of D1 (the human dose adjusted for rat metabolism) both in male (*n* = 4) and female (*n* = 4). The CAPeo doses were given (once) to animals by gavage and blood samples were collected at time 0 and 24 h. Data are represented as mean ± s.d. Asterisk denotes statistical significance (*t*-test), *p* < 0.05.

**Fig. 2:**
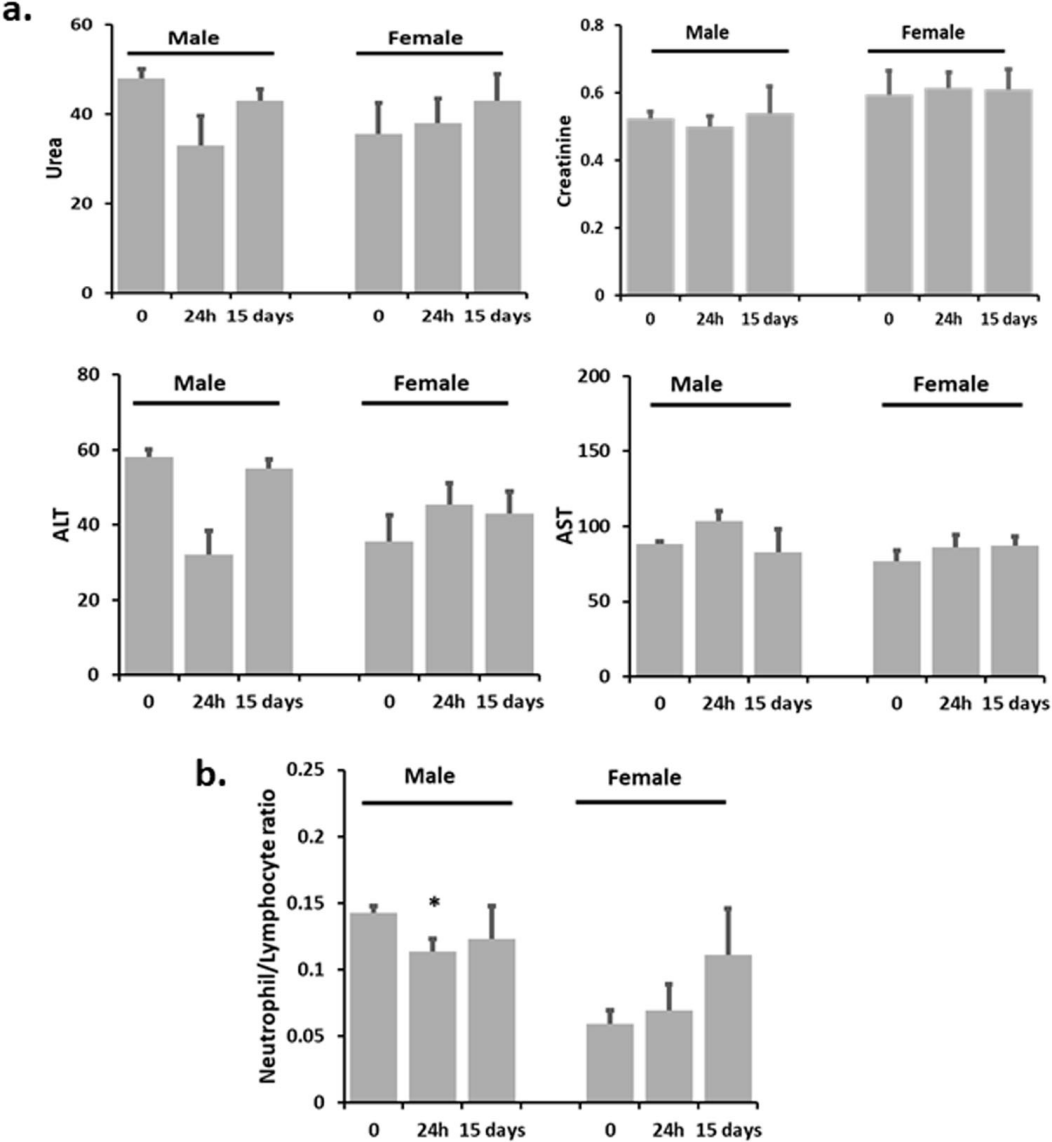
Acute effect of a high dose of CAPeo -Changes 15 days after treatment. The effect of a high dose of CAPeo on **a** creatinine (mg/dl), urea (mg/dl), AST(U/L), and ALT(U/L) levels, and **b** the neutrophil to lymphocyte ratio was examined 15 days after treatment. The CAPeo dose (40X of D1 the human dose adjusted for rat metabolism) was given (once) to animals (*n* = 2/sex) by gavage, and blood samples were collected at time 0 and 15 days. Data are represented as mean ± s.d. Asterisk denotes statistical significance (*t*-test), *p* < 0.05.

All hematological and biochemical markers assayed (at the different time points and at the end of 24 h period) in all animals treated with CAPeo were not modified compared to their values at time point zero and to only oil treated animals. Especially as it is shown in Fig. 1a none of the CAPeo doses, administered to the animals (both males and females) for 24 h, modified significantly the levels of the biochemical markers of liver and kidney toxicity (AST, ALT, creatinine, and urea), or the ratio of neutrophils/lymphocytes (N/L) calculated as a marker of inflammatory response. The increase in hepatic enzymes observed in female animals at the dose of 10X was not considered significant, as it was not confirmed in the higher dose (40X). Similarly, no modifications of the biochemical markers of liver and kidney toxicity were observed 15 days after the highest dose was given (Fig. 2). These findings indicate the absence of an acute hepato- or nephrotoxicity. On the other hand, it was found that CAPeo decreased the N/L ratio (Figs. 1b and 2b), pointing out a possible anti-inflammatory action. Interestingly, the later effect was only observed after 24 h (Fig. 2b) and only in males suggesting a sex-related effect.

In a macroscopic organ examination no detectable changes have been observed in the most important organs kidney, liver, lungs, and heart of the animals in all study groups at 24 h or 15 days. All organs examined appeared normal.

**Fig. 3:**
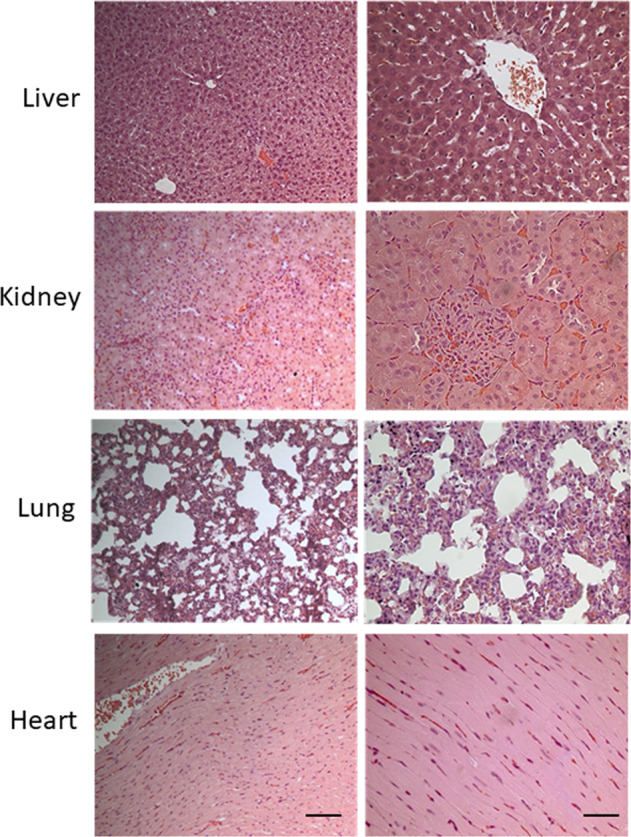
Study of CAPeo acute toxicity on selected organ tissues. Representative microphotographs of hematoxylin-eosin stained liver, kidney, lung, and heart sections of animals treated with the highest dose (40xD1). Two photos are shown for each tissue one with ×200 and one with ×400 magnification. Scale bar 100 μm and 50 μm respectively.

Finally, acute toxicity was also assessed using hematoxylin-eosin staining in formalin-fixed paraffin-embedded sections of selected tissues (kidney, liver, lungs, and heart) for all study groups. In all doses and times tested, no signs of toxicity were observed in all the organs examined after 24 h. More specifically as it is shown in Fig. 3 (presenting tissues sections from animals treated with the highest dose at 24h, as an example) no necrotic areas or any other signs of liver, kidney, lung or heart injury or inflammation were observed.

### Sub-chronic Toxicity Study

**Fig. 4:**
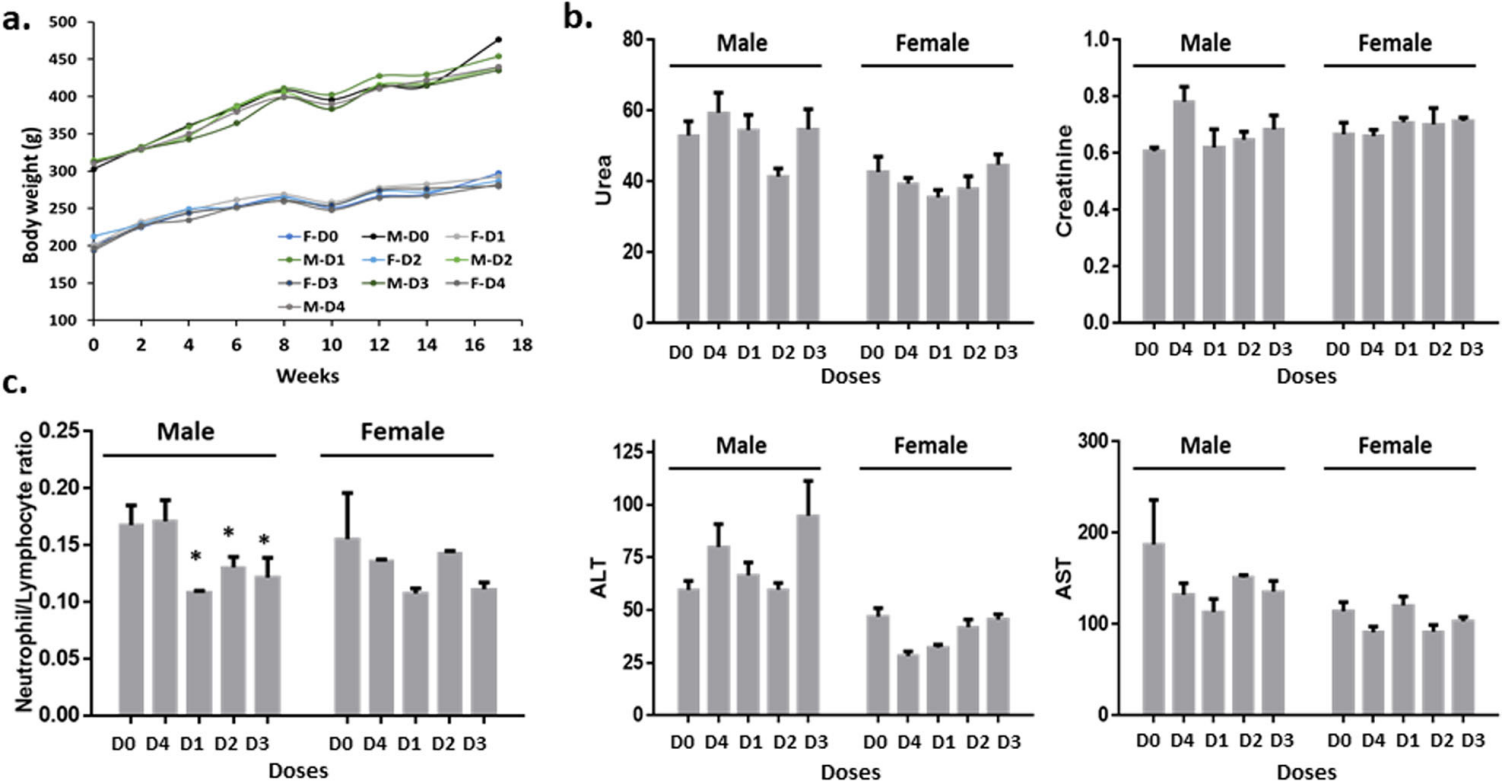
Sub-chronic effect of CAPeo. Sub-chronic CAPeo effect on **a** Body weight of the animals (M- denotes male and F- female) **b** creatinine (mg/dl), urea (mg/dl), AST(U/L), and ALT(U/L) levels, and **c** the neutrophil to lymphocyte ratio was examined. The effect of the three different CAPeo doses (D1, the human dose adjusted for rat metabolism see Methods for more details), D2: 5xD1 and D3: 20xD1) after 4 months of treatment, along with only olive oil (the vehicle-D4) and untreated animals (D0) is presented separately in male (*n* = 5) and female (*n* = 5). Data are represented as mean ± s.d. Asterisk denotes statistical significance (*t*-test), *p* < 0.05.

During the 16 weeks of the intervention, no mortality was observed, and the growth of the animals in all study groups was normal. Food intake was higher (almost double) in males than females, also reflected in their different growth rate, but no significant variations between the untreated animals, and those treated with the different doses of CAPeo were observed (Fig. 4a).

No salivation or diarrhea was noticed, and urine color was normal. Similarly, there were no changes in their skin, hair or eyes. Finally, no behavioral changes according to Irwin test or changes in food consumption and drinking activity were observed.

Several hematological and biochemical markers were assayed at the beginning and at the end of the study. During the four months of the intervention, no significant changes were observed between the different study groups. Similarly, to the acute study, neither the levels of biochemical markers of liver and kidney toxicity (AST, ALT, creatinine, and urea) nor the neutrophils/lymphocytes (N/L) ratio, being a marker of inflammation (Fig. 4), were significantly modified by any CAPeo dose, both in male and female animals. These findings indicate the absence of any sub-chronic hepato- or nephrotoxicity. Interestingly, as far as the N/L ratio is concerned, the decrease that was observed in male animals after 24 hours in the acute study it was also observed after 4 months of treatment (Fig. 4c) and only in male animals. This effect was also persistent even when findings were corrected for the differences in food consumption or growth rate between males and females.

Examination of the organs, after 4 months of daily treatment of the animals, none of the CAPeo doses induced any detectable organ changes. All organs examined had a normal size and no macroscopical alterations were noticed.

**Fig. 5:**
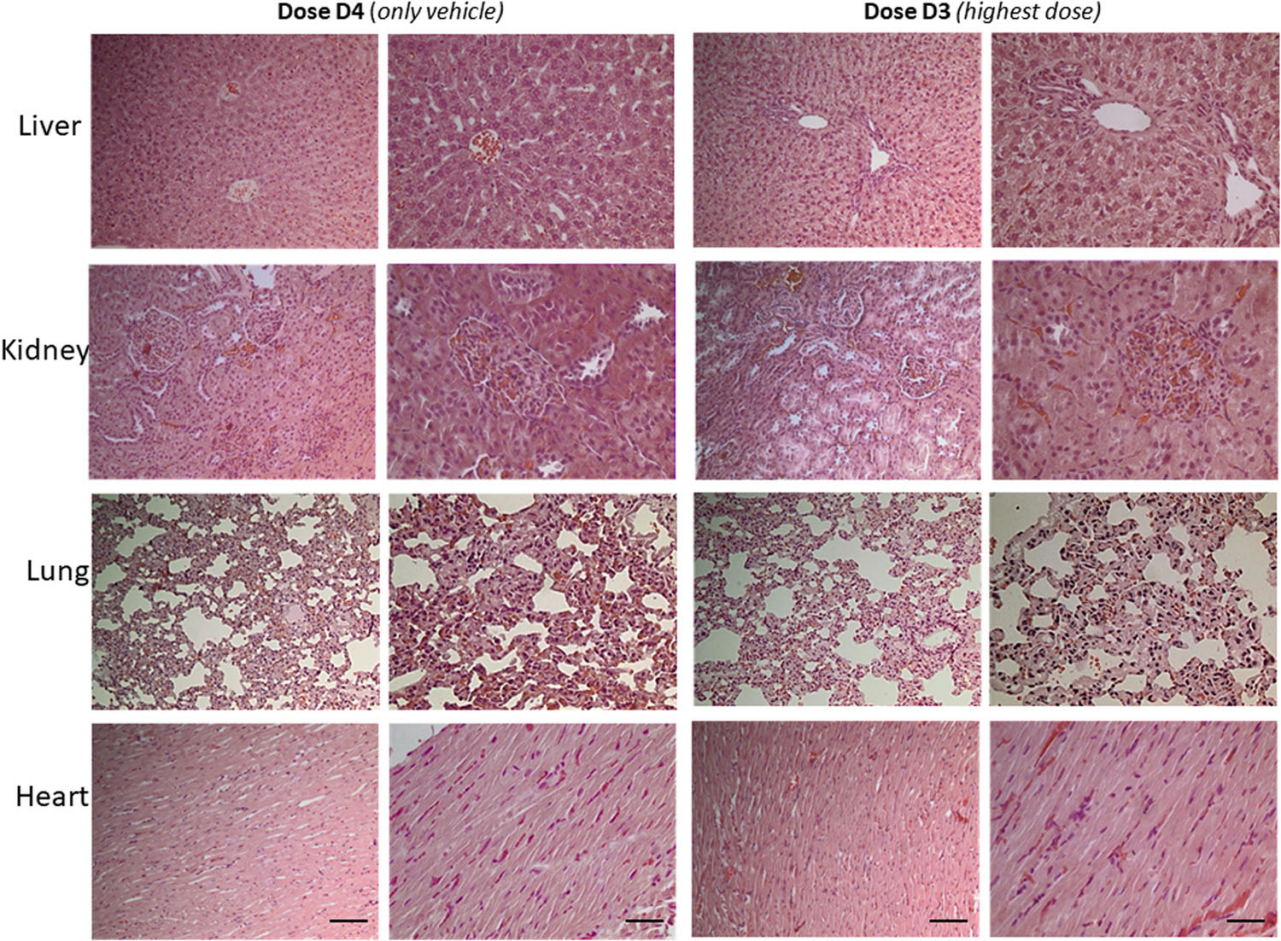
Study of CAPeo sub-chronic toxicity on selected organ tissues. Representative microphotographs of hematoxylin-eosin stained liver, kidney, lung, and heart sections of animals from the highest dose group (D3) and the only vehicle group (D4). Two photos are shown for each group and each tissue one with ×200 and one with ×400 magnification. Scale bar 100 μm and 50 μm respectively.

Hematoxylin-eosin staining of tissue sections of all organs examined did not reveal any signs of toxicity. More specifically as it is shown in Fig. 5 no necrotic or inflammatory areas or any other signs of liver, kidney, lung or heart injury were evidenced. Minimal signs of inflammation were observed in the kidneys of all animals, independently of the administration of the essential oils or not, which might be attributed to the hosting conditions. Therefore, we conclude that the long-term administration of the essential oils was safe, and this reflects the absence of biochemical toxicity, as described above.

## Discussion

Nowadays, the quest for new, more effective, and safer therapeutic remedies resulted in incorporating the ethnopharmacological and traditional knowledge and exploring the usage of several plant and herbal extracts. As a result, many therapeutic agents have been developed. Under this scope, CAPeo (Cretan Aromatic Plants essential oil) was formulated, taking advantage of previous observational studies of our group^5^, exhibiting significant antiviral properties and reducing the duration and severity of symptoms of upper respiratory tract infections^9,10^. However, since many plants can exert toxic effects, the assumption that a composition is safe because it is natural, needs careful consideration, as, in many cases, adverse reactions may occur if inappropriately used. This necessitates, along with in vitro and clinical studies, in order to confirm their efficacy, proper toxicity studies for safety validation. It should be noted that toxicity evaluation of an essential oil mixture with a specific composition is slightly different to the testing of one essential oil or a chemical, since very high doses cannot be administered in order to obtain a median lethal dose (LD_50_).

CAPeo is a mixture of essential oils of three different aromatic plants. For each of them there are either no adequate toxicity data available, such as for dittany, or the existing data discuss different species, as in the case of sage and thyme. Nevertheless, it was reported that *Thymus vulgaris* has an LD_50_ of 2840 mg/kg bw^16^ or 1000 mg/Kg bw^12^, which classifies it as moderately or slightly toxic (category 4 (LD_50_: 300–2,000 mg/kg bw), or 5 (LD_50_: 2,000–5,000 mg/kg bw) respectively, in accordance with the Globally Harmonized Classification System (GHS) established by the OECD). Similarly, for sage, data are only available for *Salvia officinalis* and not for *Salvia fruticosa* that was included in CAPeo. The LD_50_ of S. officinalis oil (when consumed orally) was 2600 mg/Kg bw^17^ classifying it as slightly toxic. There are also evidences for the major constituents of thyme, thymol and carvacrol. It seems that carvacrol is not toxic^18^, while thymol is moderately toxic^11,13,16^. Considering the major constituents of CAPeo (as presented in Table 1), it is evident that its most abundant constituents are carvacrol (52%) and eucalyptol (12%), with the latter being slightly toxic (LD_50_: 2480 mg/kg^19^, category 5). Additionally, for other less abundant constituents of CAPeo such as p-cymene^19^, β-caryophyllene^20,21^, γ-terpinene^22^, Borneol^23^ and α-Terpineol^24^, no significant toxicity has been reported (LD_50_ higher than 2000 mg/kg body weight), with the exception of α- and β-Thujone which are compounds classified at category 3 (LD_50_: 50–300 mg/kg bw)^25,26^. However, this latter compound is present in very low concentrations in CAPeo (0.74 and 0.52% cis- and trans-thujone respectively^9^, making improbable any toxicity considerations, as verified here. Moreover, the aforementioned compounds apart from α- and β-Thujone are approved by FDA and EFSA for food use (Code for Federal Regulation: 21CFR172.515 and (EU) No 872/2012 of 1 October 2012). While for α- and β-Thujone an exposure up to 7 mg/day does not pose special concerns^27^.

**Table 1:** Chemical and percentage composition of CAPeo^a^.

No	Compound	Percentage (%) composition
1	β-Myrcene	0.21
2	p-Cymene	1.32
3	Eucalyptol	12.77
4	γ-Terpinene	1.17
5	cis-Sabinene hydrate	0.45
6	trans-Sabinenhydrate	0.22
7	Linalool	0.71
8	cis-Thujone	0.74
9	trans-Thujone	0.52
10	Camphor	0.81
11	δ-Τerpineol	0.35
12	Borneol	1.68
13	Terpinen-4-ol	0.61
14	α-Terpineol	1.06
15	Thymol	0.43
16	Carvacrol	52.57
17	δ-Terpinyl acetate	0.35
18	β-Caryophyllene	3.41
19	α-Caryophyllene	0.3
20	Methylparaben	2.91
21	Caryophyllene oxide	0.45
22	Viridiflorol	0.23
23	n-Hexadecanoic acid	0.82
24	Oleic Acid	11.41
25	Squalene	4.15

^a^Table was extracted from data presented in reference^9^.

These data suggest a possible absence of toxicity for CAPeo. However, it was important to evaluate the toxicity of the CAPeo mixture itself especially at a dose range similar to those administered in humans. It is to note, that the registered use of CAPeo is for one week (1 ml/day containing ~1.5% of essential oils mixture).

In the present study, we present evidence that in an acute (24 h or 15 days) oral toxicity study CAPeo is indeed not toxic (2 different doses were tested: 10x and 40x of the effective dose in humans, modified as per the animal metabolism^28^. A higher dose could not be tested, due to the nature of CAPeo and the limited volume that can be given to the rat stomach). Similarly, no signs of toxicity were observed in sub-chronic oral administration for 4 months of the effective dose (1x), and a 5x and a 20x of this dose, also modified as per the increased rat metabolism. It should be noted that in the case of a beverage with these aromatic plants the doses examined correspond up to 80 and 40 cups per day in the acute and sub-chronic study respectively. All the evaluated parameters (appearance, behavior, complete blood cell count, biochemical and histological) were within the normal range in all doses and time periods examined. Interestingly, the neutrophil to lymphocyte ratio both in the acute and in the sub-chronic study was decreased only in male animals. This is an interesting finding which cannot be attributed to the different male and female growth and food consumption rate since in females no effect was observed even at the highest dose (corresponding to about 2 times the dose D2 in males, in which dose significant change of the neutrophil to lymphocyte ratio was observed), and points out the possibility for a sex-dependent anti-inflammatory action of CAPeo.

**Fig. 6:**
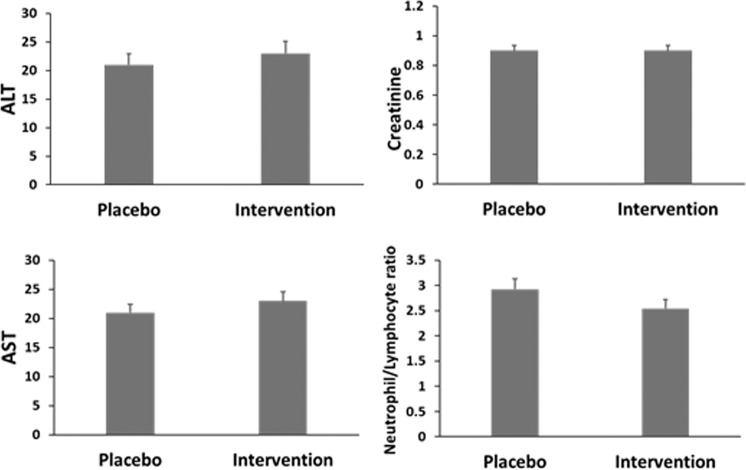
Effect of CAPeo on human biochemical parameters. CAPeo effect on creatinine (mg/dl), AST(U/L), ALT(U/L) levels, and the neutrophil to lymphocyte ratio after 7 days of intervention is presented. Analysis of data reported in Supplemental Table 6 of Duijker et al.^9^.

These animal toxicity data greatly support our previous evidence of the absence of short-term toxicity for CAPeo from the double-blind randomized controlled clinical study, where participants received CAPeo for 7 days^9,10^. Analyzing the same hematological and biochemical parameters (Fig. 6) no significant changes in the levels of biochemical markers of liver and kidney toxicity (AST, ALT, creatinine) or the ratio of neutrophils to lymphocytes, as a marker of inflammation, were observed between the intervention and the placebo group.

In conclusion, as scientific evidence of the health effects of herbal beverages is mounting, along with their popularity for daily use, it is important to have a clear and scientifically robust analysis of the safety issues involved. Here we also present, for the first time, the absence of toxicity for Cretan dittany and demonstrate that a mixture of essential oils from Cretan dittany, Greek sage and thyme can be safely utilized.

## Methods

### CAPeo composition

CAPeo is a mixture of essential oils derived from thyme or Spanish oregano (*Coridothymus capitatus* (L) Rchb. F. synonym of *Thymbra capitata* (L) Cav.), dittany or Cretan dittany (*Origanum dictamnus* L) and Greek sage (*Salvia fruticosa* Mill.). The extracts were prepared through steam distillation and analyzed by Gas Chromatography–Mass Spectroscopy. A Shimadzu, QP 5050 A apparatus with an MDN-5 column (length 30 m, film thickness 0.25 µm, diameter 0.25 mm, max. useable temperature 325 °C) and a Quadrupole Mass Spectrometer as detector were used. The temperature of the column was initially 50 °C for 5 min, gradually increased to 150 °C (5 °C/min) for 10 min, and finally increased to 280 °C (5 °C/min) and held for 20 min. The carrier gas was helium and the flow rate 0.9 mL/min. The sample injection volume was 2 μL and was measured in a split mode procedure (split ratio 1:35). The temperature for injector and detector was at 230 and 250 °C, respectively. Finally, an electron ionization system (ionization energy at 70 eV) was utilized for GS–MS detection. The chemical and percentage composition of CAPeo is presented in Table 1. For more details please refer to Supplemental Table 4 of Duijker et al.^9^.

### Acute Toxicity Study

Sprague-Dawley male and female rats (24 weeks old, weighing 300-400 gr), purchased from ENVIGO, were used (*n* = 4). This number of animals attains a statistical significance of at least *p* < 0.05, according to the web resource http://www.biomath.info/power/chsq1gp.htm). In each animal, a single dose of CAPeo essential oils, in a minimal volume of olive oil (0.25 ml) was given by gavage, directly to the stomach. The effect of two different doses was examined (10 and 40 times the corresponding dose of humans, as we have previously tested^9^, adjusted for rat metabolism^28^, D1: 0.0258 ml of 1.5% CAPeo in olive oil) both in male and female rats (see Table 2). The study was conducted according to the OECD guidelines for testing acute oral toxicity^29^. Higher doses could not be tested, due to the limitation of the volume of CAPeo (which is a mixture of essential oils in oil) that could be given to the limited volume of the rat stomach, in a single dose. The animals were single caged, had unlimited access to food and water and were kept under normal laboratory conditions. They were closely monitored for 24 h and blood samples were collected at different time points (2, 6 and 24 h) for complete blood cell counting, and biochemical analysis. Additionally, animals (male and female, *n* = 2/sex) treated by gavage with the highest dose (40 times D1) were closely monitored for 15 days and blood samples and tissues were collected at the end of the observational period.

**Table 2:** Animal numbers and doses in Acute Toxicity Study at 24 h.

Gender	Number of animals	Dose
Male	4	0 (only oil)
	4	10x D1
	4	40x D1
Female	4	0 (only oil)
	4	10x D1
	4	40x D1

*D1* 0.0258 ml CAPeo (conc: 1,5% in extra virgin olive oil).

After 24 h or 15 days animals were sacrificed, all organs were evaluated and selected tissues (kidney, liver, lungs and heart) were formalin-fixed and a detailed histological analysis was performed, for signs of inflammation, histological changes or necrosis, after Hematoxylin-Eosin staining.

### Sub-chronic Toxicity Study

Male and female Sprague-Dawley rats (8 weeks old, weighting 200–300 gr), were purchased from ENVIGO. Animals were caged in groups of 4–5 rats, had unlimited access to food and water and were kept under normal laboratory conditions. The study was conducted based on the OECD guidelines for testing sub-chronic oral toxicity^30^. Based on previous toxicity data for some of the CAPeo constituents, no toxicity and deaths were expected and therefore (taking also in to account the 3Rs for animal welfare) 10 animals per group were used in order to attain statistical significance of at least *p* < 0.05, according to the web resource http://www.biomath.info/power/ttest.htm). Animals were randomly assigned in 5 study groups for each gender separately (see Table 3): 1) No treatment group: normal diet, **D0** 2) **D1** group: 0.0258 ml of 1.5% CAPeo in olive oil/day, the dose administered to humans adjusted for rat metabolism, 3) **D2** group: 5 times Dose 1, 4) **D3** group: 20 times Dose 1, 5) **D4** group: Olive oil-Only treatment (vehicle). Due to the nature of CAPeo mixture a higher dose could not be administered.

**Table 3:** Animal numbers and doses in Sub-chronic Toxicity Study.

Group	Gender	Animal number	Dose
D0	Male	5	0 (No treatment)
	Female	5	
D1	Male	5	D1
	Female	5	
D2	Male	5	5x D1
	Female	5	
D3	Male	5	20x D1
	Female	5	
D4	Male	5	OO (only oil)
	Female	5	

*D1* 0.0258 ml CAPeo/animal /day (conc: 1.5% in extra virgin olive oil).

All animal diets were in a paste form so that olive oil and CAPeo dose could be better incorporated. All doses (see Table 3) were given adjusted in 15 ml olive oil/Kg diet. Rats were kept on these diets for 4 months. During this period, animals were closely monitored, and their food consumption and weight was recorded weekly. At the start and at the end of the study, blood samples were collected. After 4 months, animals were sacrificed, all organs were evaluated and selected tissues (kidney, liver, lungs and heart) were formalin-fixed and a detailed histological analysis was performed by two independent trained pathologists, for signs of inflammation, histological changes or necrosis, after Hematoxylin-Eosin staining.

### Ethics

Both studies were approved by the University of Crete, School of Medicine Committee for animal welfare (Protocol no. 276957) and all experiments were performed in accordance with relevant guidelines and regulations.

### Macroscopic evaluation

Animals in both studies were closely observed for mortality and any visible signs of morbidity and mortality along with changes in behavior and physiological function. More specifically according to Irwin test, their mood, perception, motor activity, and central nervous system excitation were assessed^31^. In parallel any signs of depression, salivation, diarrhea and any skin, fur and eye mucosa changes were recorded.

### Blood sample analysis

During the studies and at the end, blood samples were collected, (from rat tail vein and cardiac puncture respectively). Blood was collected in an EDTA-containing tube, gently inversed and analyzed within 2 hours after collection, for complete blood cell counting. For biochemical analysis, blood was centrifuged and serum samples were either analyzed within 3 hours or aliquoted and kept at −80 °C.

*Complete blood cell count* was performed at the University Hospital of Heraklion, Laboratory of Haematology, in Beckman-Coulter DxH600 CBC analyzer (Beckman-Coulter, CA, USA) analyzer, according to standard operating procedures.

#### Biochemical markers

All biochemical parameters (glucose, triglycerides, total cholesterol, LDL cholesterol, HDL cholesterol, hs-CRP, urea, creatinine, γGT, SGOT, and SGPT) were measured using Olympus AU2700 Analyzer at the Laboratories of Biochemistry and Clinical Immunology of the University Hospital of Heraklion.

### Pathology

For each animal a detailed gross necropsy was conducted. This included careful examination of the external surface of the body, all orifices, and the cranial, thoracic and abdominal cavities and their contents.

All organs were examined macroscopically and tissue samples were formalin-fixed (10%) and embedded in paraffin. In selected organs (liver, kidney, heart and lung), for all animals in the study groups, any signs of necrosis, injury or inflammation were assessed using hematoxylin-eosin staining. Histological analysis and evaluation were estimated by two independent pathologists.

### Statistical analysis

Statistical analysis (t-test, ANOVA, as appropriate) was performed by the GraphPad Prism V 6.0.

## Data Availability

The authors declare that all data supporting the findings of this study are available within the paper. Raw data that support the findings of this study are available from the corresponding author upon reasonable request.
